# Protective Effect of* Phaleria macrocarpa* Water Extract (Proliverenol) against Carbon Tetrachloride-Induced Liver Fibrosis in Rats: Role of TNF-*α* and TGF-*β*1

**DOI:** 10.1155/2018/2642714

**Published:** 2018-12-02

**Authors:** Nanik Sundari, Vivian Soetikno, Melva Louisa, Bantari W. Wardhani, Raymond R. Tjandrawinata

**Affiliations:** ^1^Biomedical Science, Faculty of Medicine, University of Indonesia, Jakarta, Indonesia; ^2^Department of Pharmacology and Therapeutics, Faculty of Medicine, University of Indonesia, Jakarta, Indonesia; ^3^Dexa Laboratories of Biomolecular Sciences, Dexa Medica, Jakarta, Indonesia

## Abstract

*Phaleria macrocarpa* is one of the Indonesian herbal plants which has been shown to have a hepatoprotective effect. This study was conducted to evaluate the protective effect of water extract of mahkota dewa (*Phaleria macrocarpa*) in liver fibrosis and to elucidate its mechanism of action. Male Sprague-Dawley rats were treated with carbon tetrachloride (CCl_4_) for 8 weeks to induce liver fibrosis. Rats were randomly divided into 6 groups (n=5), i.e., control group, CCl4 group, CCl4 + NAC group, CCl4 + various doses of water extract of* Phaleria macrocarpa* (50, 100, and 150 mg/kg body weight). Aspartate aminotransferase (AST), alanine aminotransferase (ALT), alkaline phosphatase (ALP), liver histopathology, malondialdehyde (MDA), ratio GSH/GSSG, Tumor Necrosis Factor- (TNF-) *α*, and Transforming Growth Factor- (TGF-) *β*_1_ were analyzed. This study demonstrated that water extract of* Phaleria macrocarpa* and NAC significantly protected CCl_4_-induced liver injury as demonstrated by reduced AST, ALT, ALP, and fibrosis percentage compared with the CCl_4_-only group. In addition, water extract of* Phaleria macrocarpa* and NAC significantly reduced the levels of MDA, TNF-*α*, and TGF-*β*_1_ as well as increasing the ratio of GSH/GSSG. Water extract of* Phaleria macrocarpa* prevents CCl_4_-induced fibrosis in rats. The prevention of liver fibrosis was at least in part through its antioxidant and anti-inflammatory activities and through its capacity to inhibit hepatic stellate cells (HSC) activation by reducing fibrogenic cytokine TGF-*β*_1_.

## 1. Introduction

Liver fibrosis is the accumulation of extracellular matrix, or excess connective tissue, in response to chronic liver injury. The main causes of liver fibrosis were viral hepatitis, alcoholic liver disease, nonalcoholic fatty liver disease (NAFLD), autoimmune disease, or hypoxia [1–4]. The progression of liver fibrosis often develops into irreversible cirrhosis and is associated with liver cancer [4].

Carbon tetrachloride (CCl_4_), a well-known hepatotoxin, is widely used in laboratory animals to induce liver injuries including liver fibrosis. CCl_4_ requires biotransformation to produce free radicals that eventually lead to membrane lipid peroxidation and causes molecular damage in hepatocytes [5, 6]. Hepatic stellate cells (HSC), whose main function is to store vitamin A and plays a pivotal role in activating the immune response via secretion of cytokines and chemokines, are activated as a result of liver injury. Once activated, HSC increase their production of extracellular matrix (ECM) protein leading to liver fibrosis. Activation of HSC is dependent on cytokines, particularly inflammation cytokines, TNF-*α*, and fibrogenic cytokines, TGF-*β* [2, 7].

*Phaleria macrocarpa *(Scheff.) Boerl., typically known as mahkota dewa, originated from Papua Island, Indonesia. Mahkota dewa is rich in alkaloid, flavonoid, and polyphenol and these compounds have pharmacological activities such as antiinflammation, antioxidant, anticancer, antidiabetic, antihypertension, and hepatoprotective [8–11].

The purpose of this study was to investigate the preventive effect of water extract of mahkota dewa fruits (Proliverenol) in CCl_4_-induced liver fibrosis in rats and its mechanism of action. For comparison we used N-acetylcysteine (NAC) which has antioxidant and free radical scavenging action [6, 12, 13].

## 2. Materials and Methods

### 2.1. Animals

Thirty male Sprague-Dawley rats weighing 200-350 g were used after 1 week for acclimatization to the animal house conditions (12-hour light/dark cycle). They were obtained from the National Agency of Drug and Food Control, Indonesia. Water and food were provided ad libitum. This study was approved by the Institutional Ethics Committee, Faculty of Medicine, Universitas Indonesia.

### 2.2. Water Extract of Mahkota Dewa Fruits (Proliverenol)

Aqueous extracts of mahkota dewa fruits (Proliverenol) were kindly provided by Dexa Laboratories of Biomolecular Sciences, PT Dexa Medica Pharmaceutical Company (Jakarta, Indonesia). The extraction method was subcritical water extraction, using water under external pressurization above its boiling point as an extraction solvent [14]. As described by Kim et al. (2010), the extraction process was conducted at 373 K, 4.0 MPa, and 5 hours [14]. The extract was then filtrated, concentrated, dried in a conventional oven, and then stored in a well closed container. Identification of Proliverenol was done by thin layer chromatography plate using ethyl acetate: acetone: formic acid: water (8: 2: 1: 1, v/v) as the eluent [15].

### 2.3. Chemicals

The chemicals used were as follows: N-acetylcysteine (NAC) (Fluimucil® oral solution 200 mg, Zambon SpA, Italy), carbon tetrachloride (Merck), PBS pH 7.4 (Sigma-Aldrich Cat. No. P4417), protease inhibitor cocktail (Boehringer Cat. No. 1697498), Kit Coomassie Plus™ (Bradford) assay (Thermo Scientific, USA, Cat. No. 23236), Kit Enzimatik ALT, AST, and ALP (Diasys, Germany), standard MDA (Aldrich 10838-3), trichloroacetic acid (TCA) (Merck K40385207), Oxyselect™ Total Glutathione (GSSG/GSH) (Cell Biolabs Cat. No. STA-312), standard GSH (Sigma-Aldrich 66529), ELISA kit TNF-*α* (Sigma-Aldrich Cat. No. RAB0480), and ELISA kit TGF-*β*1 (Novateinbio Cat. No. FM-E100129).

### 2.4. Experimental Design

The rats were randomly allocated to the six groups, namely, the control group (n = 5), the CCl_4_ group (n = 5), the CCl_4_ + NAC group (n = 5), the CCl_4_ + Proliverenol 50 mg/kg BW group (n = 5), the CCl_4_ + Proliverenol 100 mg/kg BW group (n = 5), and the CCl_4_ + Proliverenol 150 mg/kg BW group (n = 5). Proliverenol and NAC were dissolved in olive oil. The model of liver fibrosis was induced by i.p. injection with CCl_4_ (0.2 mL/100 g BW) mixed with olive oil as vehicle in 1:1 ratio twice weekly for 2 weeks followed by i.p. injection of mixed CCl_4_ (0.1 mL/100 g BW) and olive oil (1:1 v/v) twice weekly for 6 weeks as described by Constandinou [16]. NAC and Proliverenol were given once daily by oral gavage for 8 weeks.

Seventy-two hours after the last CCl_4_ injection, rats were sacrificed, and blood samples were collected in the EDTA tubes and centrifuged at 3,000* g*, 4°C for 15 min, to obtain plasma. The samples were stored at -20°C until analyzed. The liver was excised from each animal, and a portion of the liver was taken for histopathological examination, while the remaining tissue was kept in freezer at -80°C until analyzed.

### 2.5. Biochemical Analysis

Activities of ALT, AST, and ALP in plasma and the ratio of GSH/GSSG in the liver were determined according to the manufacturer's guideline. Hepatic lipid peroxidation was determined as thiobarbituric acting-reacting substance and is expressed as equivalent of malondialdehyde (MDA) using 1,1,3,3-tetramethoxypropane as standard. Levels of TNF-*α* and TGF-*β*_1_ in the liver were determined using ELISA kits according to the manufacturer's guideline. Protein concentrations were measured according to the manufacturer's guideline.

### 2.6. Liver Histopathological Analysis

Liver tissue samples were fixed in 10% formalin, embedded in paraffin, sectioned, and stained with Masson's trichrome to identify increases in liver collagenous tissue. The degrees of liver fibrosis were evaluated by a pathologist from the Faculty of Veterinary Medicine, Bogor Agricultural University, in a blinded manner. Quantification of the degree of fibrosis was done by ImageJ software with threshold-color plug-in logarithm. Percentage of fibrosis was calculated by dividing the area of fibrosis (blue regions) by the total area.

### 2.7. Statistical Analysis

SPSS 16.0 software package was used for statistical analysis. Results were expressed as means ± standard deviations (SD). Statistical analysis was performed by one-way ANOVA test followed by Tukey's postanalysis test for multiple comparisons.* p* <0.05 was considered as statistically significant.

## 3. Results

### 3.1. Biochemical Markers

**Figure 1:**
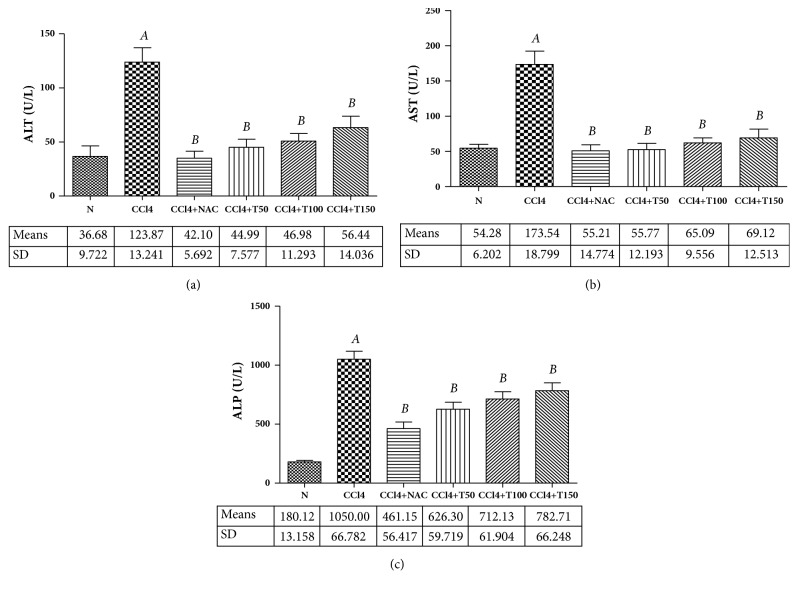
*(a) Plasma ALT levels of normal control, CCl*_4_ *, CCl*_4+_ *NAC150, CCl*_4+_ *T50, CCl*_4+_ *T100, and CCl*_4+_ *T150 groups.* Data represent the means ± SD (n=5). N: normal, CCl_4_: carbon tetrachloride, NAC: N-acetylcysteine 150 mg/kg body weight/day, and T50, T100, and T150: Proliverenol 50, 100, and 150 mg/kg body weight/day.* A *=* p* < 0.05 vs. N,* B *=* p* < 0.05 vs. CCl_4_ (*α*=0.05).* (b) Plasma AST levels of normal control, CCl*_4_*, CCl*_4+_*NAC150, CCl*_4+_*T50, CCl*_4+_*T100, and CCl*_4+_*T150 groups.* Data represent the means ± SD (n=5). N: normal, CCl_4_: carbon tetrachloride, NAC: N-acetylcysteine 150 mg/kg body weight/day, and T50, T100, and T150: Proliverenol 50, 100, and 150 mg/kg body weight/day.* A *=* p* < 0.05 vs. N,* B *=* p* < 0.05 vs. CCl_4_ (*α*=0.05).* (c) Plasma ALP levels of normal control, CCl*_4_*, CCl*_4+_*NAC150, CCl*_4+_*T50, CCl*_4+_*T100, and CCl*_4+_*T150 groups.* Data represent the means ± SD (n=5). N: normal, CCl_4_: carbon tetrachloride, NAC: N-acetylcysteine 150 mg/kg body weight/day, and T50, T100, and T150: Proliverenol 50, 100, and 150 mg/kg body weight/day.* A *=* p* < 0.05 vs. N,* B *=* p* < 0.05 vs. CCl_4_ (*α*=0.05).

Plasma ALT, AST, and ALP activities were measured as markers of liver injury. The effect of oral administration of Proliverenol and NAC on plasma ALT, AST, and ALP activities is presented in Figures 1(a)–1(c). Significant increases in the activities of these marker enzymes were observed in CCl_4_-treated rats. Treatment with Proliverenol and NAC significantly decreased those enzyme activities.

### 3.2. Histopathological Findings

**Figure 2:**
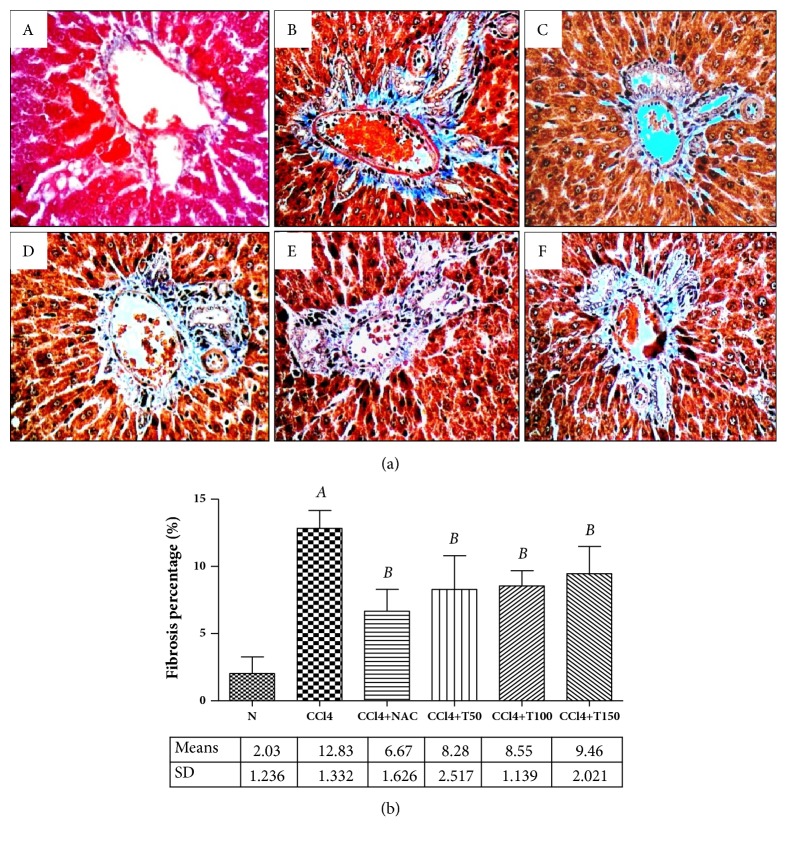
*(a) The liver histopathological examination samples stained by Masson's trichrome (400x).* Blue color showed collagen deposition around portal triad. A: normal group. B: CCl_4_ group. C: CCl_4_+NAC group. D: CCl_4_+T50 group. E: CCl_4_+T100 group. F: CCl_4_+T150 group. N: normal, CCl_4_: carbon tetrachloride, NAC: N-acetylcysteine 150 mg/kg body weight/day, and T50, T100, and T150: DLBS Proliverenol 50, 100, and 150 mg/kg body weight/day.* (b) Fibrosis percentage of normal control, CCl*_4_*, CCl*_4+_*NAC150, CCl*_4+_*T50, CCl*_4+_*T100, and CCl*_4+_*T150 groups.* Data represent the means ± SD (n=5). N: normal, CCl_4_: carbon tetrachloride, NAC: N-acetylcysteine 150 mg/kg body weight/day, and T50, T100, and T150: Proliverenol 50, 100, and 150 mg/kg body weight/day.* A *=* p* < 0.05 vs. N,* B *=* p* < 0.05 vs. CCl_4_ (*α*=0.05).

Histopathological examinations of the liver sections and the percentage of liver fibrosis were presented in Figures 2(a) and 2(b). Significant increases of liver fibrosis were observed in CCl_4_-treated rats. Interestingly, treatment with Proliverenol and NAC significantly decreased the percentage of liver fibrosis.

### 3.3. Hepatic MDA and Ratio GSH/GSSG

**Figure 3:**
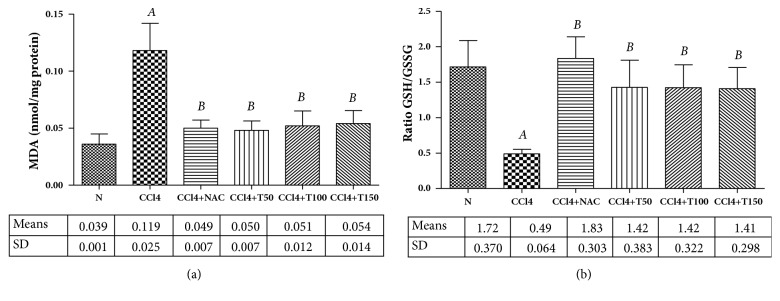
*(a) Liver MDA levels of normal control, CCl*_4_ *, CCl*_4+_ *NAC150, CCl*_4+_ *T50, CCl*_4+_ *T100, and CCl*_4+_ *T150 groups.* Data represent the means ± SD (n=5). N: normal, CCl_4_: carbon tetrachloride, NAC: N-acetylcysteine 150 mg/kg body weight/day, and T50, T100, and T150: Proliverenol 50, 100, and 150 mg/kg body weight/day.* A *=* p* < 0.05 vs. N,* B *=* p* < 0.05 vs. CCl_4_ (*α*=0.05).* (b) Ratio GSH/GSSG of normal control, CCl*_4_*, CCl*_4+_*NAC150, CCl*_4+_*T50, CCl*_4+_*T100, and CCl*_4+_*T150 groups.* Data represent the means ± SD (n=5). N: normal, CCl_4_: carbon tetrachloride, NAC: N-acetylcysteine 150 mg/kg body weight/day, and T50, T100, and T150: Proliverenol 50, 100, and 150 mg/kg body weight/day.* A *=* p* < 0.05 vs. N,* B *=* p* < 0.05 vs. CCl_4_ (*α*=0.05).

Oxidative stress plays an important role in the development of liver fibrosis. MDA, the main final product of lipid peroxidation, was measured in rats' livers to determine the membrane lipid oxidative damage [17], whereas the ratio of GSH/GSSG was measured as an indicator of oxidative stress [17, 18]. A significant increase of MDA levels and a significant decrease of GSH/GSSG ratio were observed in CCl_4_-treated rats, while the levels of MDA and the ratio of GSH/GSSG were decreased and increased, respectively, in the Proliverenol and NAC groups, as compared to the CCl_4_-treated groups (Figures 3(a) and 3(b)).

### 3.4. Hepatic Level of TNF-*α*

**Figure 4:**
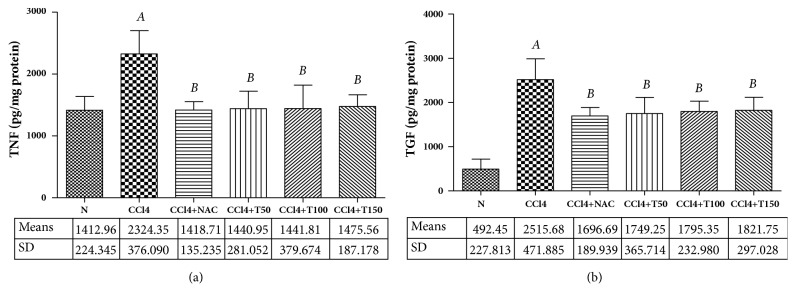
*(a) Liver TNF-α levels of normal control, CCl*_4_ *, CCl*_4+_ *NAC150, CCl*_4+_ *T50, CCl*_4+_ *T100, and CCl*_4+_ *T150 groups.* Data represent the means ± SD (n=5). N: normal, CCl_4_: carbon tetrachloride, NAC: N-acetylcysteine 150 mg/kg body weight/day, and T50, T100, and T150: Proliverenol 50, 100, and 150 mg/kg body weight/day.* A *=* p* < 0.05 vs. N,* B *=* p* < 0.05 vs. CCl_4_ (*α*=0.05).* (b) Liver TGF-β1 levels of normal control, CCl*_4_*, CCl*_4+_*NAC150, CCl*_4+_*T50, CCl*_4+_*T100, and CCl*_4+_*T150 groups.* Data represent the means ± SD (n=5). N: normal, CCl_4_: carbon tetrachloride, NAC: N-acetylcysteine 150 mg/kg body weight/day, and T50, T100, and T150: Proliverenol 50, 100, and 150 mg/kg body weight/day.* A *=* p* < 0.05 vs. N,* B *=* p* < 0.05 vs. CCl_4_ (*α*=0.05).

Inflammation is commonly associated with liver fibrosis during chronic liver injury [13, 17]. To explore the mechanisms underlying the protective effect of Proliverenol on liver fibrosis, we proposed that Proliverenol might protect the liver against CCl_4_-induced injury by suppressing inflammation in the liver. The levels of proinflammatory cytokines TNF-*α* were determined in the liver by ELISA. As shown in Figure 4(a), significantly increased levels of TNF-*α* were observed in CCl_4_-treated rats. Treatment with Proliverenol and NAC decreased the levels of TNF-*α* in a significant manner.

### 3.5. Hepatic Level of TGF-*β*_1_

TGF-*β*_1_ is the major profibrogenic cytokine [1–3, 19]. To elucidate the underlying mechanisms of Proliverenol in the suppression of liver fibrogenesis, we measured the levels of TGF-*β*_1_ in the rat model. The levels of profibrogenic cytokines TGF-*β*_1_ were determined in the liver by ELISA. As shown in Figure 4(b), significantly increased levels of TGF-*β*_1_ were observed in CCl_4_-treated rats. On the other hand, treatment with Proliverenol and NAC significantly decreased the levels of TGF-*β*_1_.

## 4. Discussion

This study has demonstrated that intraperitoneal injection of CCl_4_ to rats for 8 weeks leads to a marked elevation in the activities of plasma ALT, AST, and ALP as compared with the control group. Our results were in agreement with the findings of Morsy et. al (2012) [6], Cháved E et. al (2008) [7], Demiroren et. al (2014) [13], and Fu et. al (2008) [20], in which they used CCl_4_ to develop a model of liver fibrosis. The development of liver fibrosis is a dynamic process characterized by deposition of collagen and other cellular matrix proteins, following activation of HSC into myofibroblast-like cells which resulted in the secretion of several profibrogenic cytokines such as TGF-*β*, endothelin-1, and platelet-derived growth factor [21, 22]. Treatment with Proliverenol and NAC significantly reduced the activities of those marker enzymes as compared with CCl_4_-treated rats. This result implied that Proliverenol and NAC potentially prevent liver damage by suppressing the leakage of enzymes through cellular membranes and hence restore the enzymes' activities in the hepatocytes. However, there was no evidence of a dose-response relationship with regard to efficacy parameters including fibrosis percentage, MDA, GSH/GSSG ratio, TNF-*α*, and TGF-*β* among treatments. Our results with Proliverenol are in agreement with results obtained by Nailufar et al. in ethanol-induced liver damage using DLBS1433 (Proliverenol). They showed that, among various concentrations of DLBS1433-60, maximum antioxidant activity was showed by DLBS1433-60 at a concentration of 100 ppm with no further increase of antioxidant activity if the concentration was increased [23].

The histopathological analysis revealed that chronic administration of CCl_4_ produced a marked increase in collagen deposition, as evidenced by the blue stains around the portal triad and increased the percentage of liver fibrosis. Treatment with Proliverenol and NAC significantly reduced the percentage of liver fibrosis as compared with CCl_4_-treated and control rats.

The levels of MDA were quite increased in the CCl_4_ group as compared to the control group. This result was in agreement with the finding of Pereira-Filho et. al (2008) [5], Morsy et. al (2002) [6], Cháved E et. al (2008) [7], and Kamalakkannan et. al (2005) [12]. This increase may be associated with the generation of trichloromethyl radicals (^*∙*^CCl_3_) and trichloromethyl peroxyl radical (^*∙*^O_2_CCl_3_) following metabolism of CCl_4_ by cytochrome P450 enzyme [24–26]. In this study, we demonstrated that the levels of MDA were decreased in the CCl_4_ + Proliverenol groups as well as in the CCl_4_ + NAC group. We believe that the antioxidant capacity to scavenge reactive oxygen species is the main mechanism of action of Proliverenol and NAC to protect the hepatic parenchyma against the trichloromethyl and trichloromethyl peroxyl radicals.

GSH is an important nonenzymatic antioxidant required to maintain the normal redox state of cells [17, 27]. Oxidative stress induced by CCl_4_ results in the increased utilization of GSH and subsequently decreased the ratio GSH/GSSG in tissue, thereby affecting functional and structural integrity of cell and organelle membranes [17]. Our results showed that oral administration Proliverenol and NAC with CCl_4_ increased the ratio GSH/GSSG in liver tissues as compared to CCl_4_ administered alone, which indicated their potential to counteract the oxidative damage induced by CCl_4_ and to reinforce the antioxidant defense in normal conditions.

TNF-*α* is a key factor that triggers an inflammatory cascade involving the induction of cytokine after liver injury. The injured liver, predominantly Kupffer cells and infiltrating macrophages and neutrophils, will produce TNF-*α*. In particular, TNF-*α* plays a dichotomous role in the liver, where it not only induces hepatocyte proliferation, apoptosis, and inflammation but also is known to suppress collagen *α*1 gene expression [13, 17, 20, 27, 28]. Our results showed that oral administration of Proliverenol and NAC with CCl_4_ reduced the levels of TNF-*α* in the liver as compared to CCl_4_ administered alone. This result suggested that protective effect of Proliverenol and NAC on CCl_4_-induced liver fibrosis was at least, in part, due to their capabilities to suppress TNF-*α* production.

Activation of HSC was triggered by TGF-*β*_1_, which was released from Kupffer cells as well as oxidative stress caused by CCl_4_ [16]. Besides, activation of HSC can also be provoked by a range of chronic injuries to the liver such as viral hepatitis, toxins, and autoimmune disorders [29]. TGF-*β*_1_ is a major profibrogenic cytokine. Its main functions are regulating the production, degradation, and accumulation of the ECM in liver fibrosis. TGF-*β*_1_ leads fibrogenesis through autocrine and paracrine effects of HSC [17, 20, 30]. Our study showed that oral administration of Proliverenol and NAC with CCl_4_ reduced the levels of TGF-*β*_1_ in liver tissue as compared to CCl_4_ administered alone. This suggested that protective effect of Proliverenol and NAC on CCl_4_-induced liver fibrosis was associated with their abilities to inhibit HSC activation by reducing TGF-*β*_1_ production.

In conclusion, the protective effect of Proliverenol in CCl_4_-induced liver fibrosis in rats appears to stem from its antioxidant capacities as indicated by protection against increased lipid peroxidation and decreased ratio GSH/GSSG and its ability to suppress proinflammatory cytokines TNF-*α* and to inhibit HSC activation by reducing profibrogenic cytokines TGF-*β*_1_. Thus, we believe that Proliverenol might be a therapeutic candidate for the prevention of liver fibrosis.

## Data Availability

The data used to support the findings of this study are available from the corresponding author upon request.
